# Cerebral Protein Synthesis in a Knockin Mouse Model of the Fragile X Premutation

**DOI:** 10.1177/1759091414551957

**Published:** 2014-09-19

**Authors:** Mei Qin, Tianjian Huang, Zhonghua Liu, Michael Kader, Thomas Burlin, Zengyan Xia, Zachary Zeidler, Renate K. Hukema, Carolyn B. Smith

**Affiliations:** 1 Section on Neuroadaptation and Protein Metabolism, National Institute of Mental Health, National Institutes of Health, Bethesda, MD, USA; 2 Department of Clinical Genetics, Erasmus MC, Rotterdam, The Netherlands

**Keywords:** *Fmr1*, FMRP, fragile X premutation, fragile X syndrome, FXTAS, protein synthesis

## Abstract

The (CGG)n-repeat in the 5′-untranslated region of the fragile X mental retardation gene (*FMR1*) gene is polymorphic and may become unstable on transmission to the next generation. In fragile X syndrome, CGG repeat lengths exceed 200, resulting in silencing of *FMR1* and absence of its protein product, fragile X mental retardation protein (FMRP). CGG repeat lengths between 55 and 200 occur in fragile X premutation (FXPM) carriers and have a high risk of expansion to a full mutation on maternal transmission. FXPM carriers have an increased risk for developing progressive neurodegenerative syndromes and neuropsychological symptoms. *FMR1* mRNA levels are elevated in FXPM, and it is thought that clinical symptoms might be caused by a toxic gain of function due to elevated *FMR1* mRNA. Paradoxically, FMRP levels decrease moderately with increasing CGG repeat length in FXPM. Lowered FMRP levels may also contribute to the appearance of clinical problems. We previously reported increases in regional rates of cerebral protein synthesis (rCPS) in the absence of FMRP in an *Fmr1* knockout mouse model and in a FXPM knockin (KI) mouse model with 120 to 140 CGG repeats in which FMRP levels are profoundly reduced (80%–90%). To explore whether the concentration of FMRP contributes to the rCPS changes, we measured rCPS in another FXPM KI model with a similar CGG repeat length and a 50% reduction in FMRP. In all 24 brain regions examined, rCPS were unaffected. These results suggest that even with 50% reductions in FMRP, normal protein synthesis rates are maintained.

## Introduction

The (CGG)n-repeat sequence in the 5′-untranslated region of the fragile X mental retardation gene (*FMR1*) on Xq27.3 is polymorphic. In the normal population, the repeat sequence length is 5 to 54 and the gene product, fragile X mental retardation protein (FMRP) has normal expression (Fu et al., 1991). Individuals with fragile X syndrome (FXS), the most common form of hereditary intellectual disability and monogenic cause of autism, have more than 200 CGG repeats resulting in gene methylation, transcriptional silencing, and absence of FMRP (Verkerk et al., 1991). This is known as the full mutation. Individuals with (CGG)n between 55 and 200 are known as fragile X premutation (FXPM) carriers. The gene remains unmethylated in these individuals and transcription is not silenced, but there is a high risk of expansion to a full mutation on maternal transmission. Because the prevalence of FXPM in the general population, approximately 1/290 males and 1/148 females (Maenner et al., 2013), is much higher than that of FXS, FXPM may have a greater impact on the general population than FXS.

FXPM carriers are at increased risk for developing progressive neurodegenerative syndromes, including fragile X-associated tremor/ataxia syndrome (FXTAS; Hagerman et al., 2001) and fragile X-associated primary ovarian insufficiency (FXPOI) in females (Allingham-Hawkins et al., 1999). Further evidence suggests that FXPM carriers have a higher incidence of neuropsychological symptoms (Cornish et al., 2005, 2009; Hunter et al., 2008; Kogan et al., 2008). These features are thought to be caused by a toxic gain of function of elevated levels of CGG repeat-containing *FMR1* mRNA (Jin et al., 2003; Hessl et al., 2005; Brouwer et al., 2008a; Hagerman, 2013). As FMRP levels moderately decrease with increasing CGG repeat length (Tassone et al., 2000; Peprah et al., 2010; Ludwig et al., 2014), lowered FMRP levels could also contribute to clinical symptoms (Ludwig et al., 2014).

To better understand FXPM, two expanded CGG repeat knockin (KI) models have been generated in mice (Bontekoe et al., 2001; Entezam et al., 2007). In one (KI_Dutch_), a region of the *Fmr1* gene including the endogenous repeat tract (CGG)_8_ was replaced with a cloned human premutation allele (CGG)_98_ (Bontekoe et al., 2001). The repeat tract in the other model (KI_NIH_) was generated by serial ligation of short, stable CGG·CCG repeats (Grabczyk and Usdin, 1999; Entezam et al., 2007). As in the human disease, the KI mice in both models exhibit repeat-length instabilities on transmission to succeeding generations and a direct relationship between repeat number and *Fmr1* mRNA levels in brain (Bontekoe et al., 2001; Entezam et al., 2007). KI mice also display behavioral phenotypes similar to those seen in human FXPM individuals, such as motor deficits, anxiety, and impairments of learning and memory (Van Dam et al., 2005; Hunsaker et al., 2009, 2011, 2012; Qin et al., 2011; Diep et al., 2012). FMRP levels tend to decrease with increasing CGG repeat length, and in both KI models, brain FMRP levels are decreased; the effect size varies across studies (Willemsen et al., 2003; Brouwer et al., 2007, 2008a, 2008b; Entezam et al., 2007; Qin et al., 2011; Berman et al., 2012; Ludwig et al., 2014). Previously, we studied young adult, male KI_NIH_ PM mice with CGG_(120–140)_ and found that they had a very similar behavioral phenotype to *Fmr1* knockout (KO) mice (Qin et al., 2011). We found that young adult KI_NIH_ mice exhibited hyperactivity in the open field, less general anxiety in both the open field and elevated zero maze, impaired learning and memory on a passive avoidance test, and subtle deficits on a test of social interaction. Motor learning was normal as assessed by the rotarod test. In our study, KI_NIH_ mice had two- and sixfold increases in *Fmr1* mRNA and drastic decreases in levels of FMRP; FMRP levels in whole brain were reduced to 15% of wild type (WT). Additionally, regional rates of cerebral protein synthesis (rCPS) measured *in vivo* were statistically significantly increased similar to our finding in *Fmr1* KO mice (Qin et al., 2005), suggesting that the decreased concentration of FMRP may contribute to the KI_NIH_ phenotype. Our goal in the present study was to test how the expanded CGG repeat sequence in *Fmr1* affects rCPS in a model (KI_Dutch_) in which FMRP levels are reported to be either not affected (Willemsen et al., 2003) or moderately reduced (Brouwer et al., 2008a; Berman et al., 2012).

## Materials and Methods

### Animals

The KI_Dutch_ mice generated by exchanging the murine endogenous (CGG)_8_ with a human (CGG)_98_ (Bontekoe et al., 2001) were obtained from Erasmus Medical College. The genetic background was C57BL/6. Animals for these experiments were bred in-house. Briefly, WT and KI_Dutch_ mice were produced by pairing female mice heterozygous for the KI_Dutch_ allele with WT males. This strategy yielded WT and KI_Dutch_ male mice in the same litters. Mice were group housed in a central facility and maintained under controlled conditions of normal humidity and temperature with standard alternating 12-hr periods of light and darkness. All procedures were carried out in accordance with the National Institutes of Health Guidelines on the Care and Use of Animals and an animal study protocol approved by the National Institute of Mental Health Animal Care and Use Committee.

### Determination of CGG Repeat Size

DNA was extracted from mouse tail with Gentra Puregene Mouse Tail Kit (Puregene, Gentra Systems, Inc., Minneapolis, MN, USA) and kept in Tris-EDTA (TE) buffer. The size of the CGG repeat tract was monitored by polymerase chain reaction (PCR) with the primers CGG-F (5′-CGGAGGCGCCGCTGCCAGG-3′) and CGG-R (5′-TGCGGGCGCGCTCGAGGCCCAG-3′) as described previously (Hukema and Oostra, 2013).

### Western Blotting

WT and KI_Dutch_ mice were anesthetized with sodium pentobarbital (100 mg/kg, i.p.) and decapitated. The cerebellum and cerebrum were separated and frozen in dry ice. Frozen sections, 100 µm in thickness, were prepared at −22°C (Leica 1850 cryostat; Leica Microsystems, Deerfield, IL), and 11 regions were punched by means of Harris Uni-Core (1.25 mm; Electron Microscopy Sciences, Hatfield, PA). Regions were weighed and homogenized in 1% (w/v) ice-cold tissue protein extraction reagent (T-PER) (Thermo Scientific, Rockford, IL) with 1% Halt protease inhibitor cocktail (Thermo Scientific) and 1% phosphatase inhibitor cocktail (Sigma-Aldrich, St. Louis, MO, USA). Homogenates were centrifuged (12,000 × g, 4°C, 15 min). The supernatant fractions were collected as protein samples, which were separated by NuPAGE on Bis–Tris gels and subjected to Western blotting with a WesternBreeze Chemiluminescent kit (Invitrogen, Carlsbad, CA). FMRP was detected with a rabbit polyclonal antibody to FMRP, ab17722 (Abcam Inc, Cambridge, MA). Protein extracts from the brains of *Fmr1* KO animals were used as negative controls. Blots were quantified by densitometry with MCID Analysis system (Interfocus Imaging Ltd, Linton, Cambridge, UK). Glyceraldehyde-3-phosphate dehydrogenase (GAPDH rabbit monoclonal antibody; Cell Signaling Technology, Danvers, MA) was used as a loading control.

### Regional Rates of Cerebral Protein Synthesis (rCPS)

We used the autoradiographic L-[1-^14^C] leucine method to determine rCPS in young adult WT (*n* = 7) and KI_Dutch_ (*n* = 7) mice as described previously (Qin et al., 2005). Briefly, mice under light isoflurane anesthesia were prepared for studies by insertion of polyethylene catheters into a femoral artery and vein. Mice recovered from the surgery overnight and were permitted to move freely. The experimental period was initiated by an intravenous pulse injection of 100 µCi/kg of L-[1-^14^C]leucine (specific activity, 60 mCi/mmol; Moravek Biochemicals, Brea, CA). Timed arterial samples were collected during the following 60 min for determination of the time courses of plasma concentrations of leucine and [^14^C]leucine. At the end of the experimental interval, brains were removed, frozen, and serial sections, 20 µm in thickness, were prepared for quantitative autoradiography. Autoradiograms were digitized (MCID Analysis, Interfocus Imaging Ltd, Linton, Cambridge, UK), the concentration of ^14^C in each region of interest was determined, and rCPS was calculated by means of the operational equation of the method (Smith et al., 1988). The value of lambda in the equation was 0.603 (Qin et al., 2005). Brain regions were identified by reference to a mouse brain atlas (Paxinos and Franklin, 2001).

### Statistical Analysis

Data are expressed as mean ± SEM. Statistically significant interactions were analyzed by repeated measures (RM) analysis of variance (ANOVA). Data from Western blots were analyzed for differences between WT and KI_Dutch_ by means of one-tailed Student’s *t* tests. The criterion for statistical significance was *p* ≤ .05. We used SPSS (IBM, Armonk, NY) for statistical analyses.

## Results

### FMRP Levels in Brain are Negatively Correlated With Number of CGG Repeats

**Figure 1.**
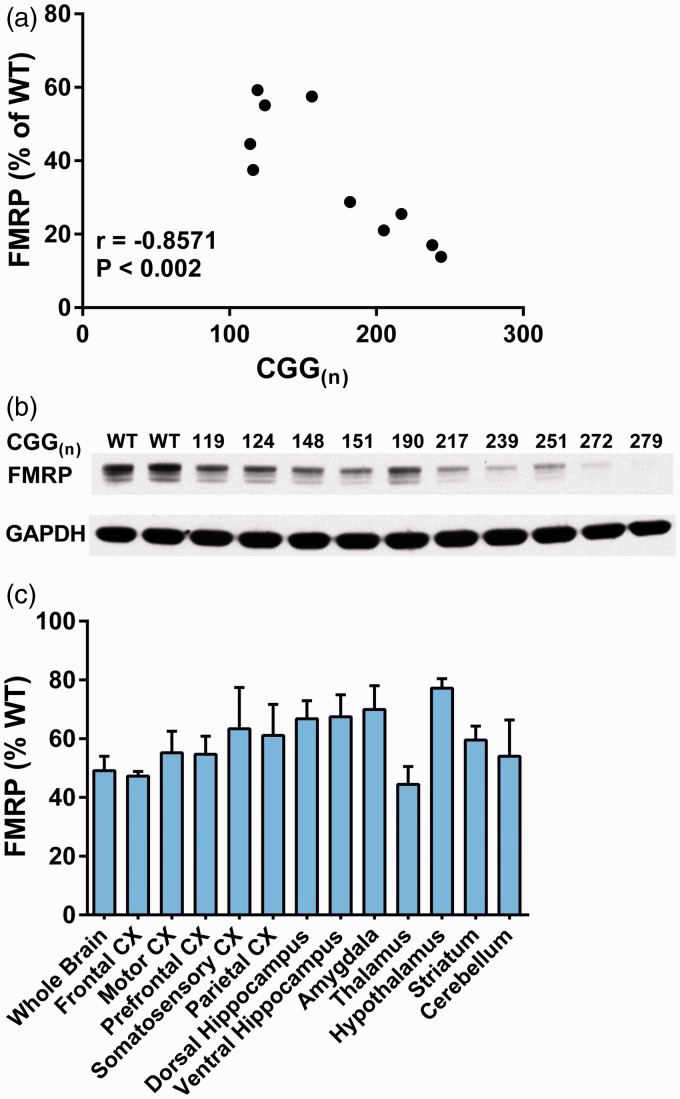
Effects of CGG repeat insertion on FMRP in brain regions. (a) Change in FMRP/GAPDH in whole brain as % of WT controls. KI_Dutch_ mice had CGG repeat lengths ranging from 119 to 279. The negative correlation (Pearson correlation coefficient, −.8571) is statistically significant (*p* < .002). The WT control FMRP/GAPDH is the mean of two determinations (0.464, 0.481). (b) Western blot of whole brain extracts from WT and KI_Dutch_ mice with CGG repeats range 119 to 279. (c) Change in FMRP/GAPDH ratios in whole brain and 12 subregions as % of WT controls. KI_Dutch_ mice had CGG repeats range 119 to 151. Bars are the mean ± SEM determined in three mice of each genotype. We used a rabbit polycolonal antibody to FMRP (ab17722). FMRP = fragile X mental retardation protein; WT = wild type; GAPDH = glyceraldehyde-3-phosphate dehydrogenase; CX = cortex.

We determined the number of CGG repeats and whole brain levels of FMRP in 10 KI_Dutch_ mice. The number of CGG repeats ranged from 114 to 279, and FMRP levels decreased from 59% to 14% of the mean of three WT values (Figure 1(a) and (b)). We found a statistically significant negative correlation between FMRP levels and the number of CGG repeats (*p* < .002). For our studies of rCPS, we used mice with CGG repeat lengths between 120 and 159 (Table 1). In mice with a similar range of CGG repeat lengths (119–151), we found that average brain FMRP levels were reduced to 49% of WT controls. We also determined FMRP levels in 12 brain regions from three KI_Dutch_ mice (CGG repeat lengths between 108 and 128) and found that levels ranged from 44% to 77% of WT controls (Figure 1(b)). Frontal association cortex and thalamus had the greatest decreases (47%, 44% of WT), and hypothalamus had the mildest decrease (77% of WT).

**Table 1.** Physiological Variables.

	Wild type (7)	Knockin (7)
Age (days)	130 ± 0.5	129 ± 0.9
Body weight (g)	31.0 ± 1.8	32.8 ± 0.7
Arterial plasma glucose concentration (mM)	5.3 ± 0.4	5.8 ± 0.3
Arterial blood hematocrit (%)	45 ± 1	44 ± 1
Mean arterial blood pressure (mm Hg)	111 ± 1	113 ± 1
CGG repeat length	ND	137 ± 6

*Note.* Physiological variables were measured at the time of rCPS study. Values are mean ± SEM for the number of mice indicated in parentheses. ND = not determined.

### Unaltered rCPS in KI_Dutch_ Mice

At the time of rCPS determination, physiological variables in WT and KI_Dutch_ mice were well matched with respect to age, body weight, and other physiological variables measured (Table 1).

**Figure 2.**
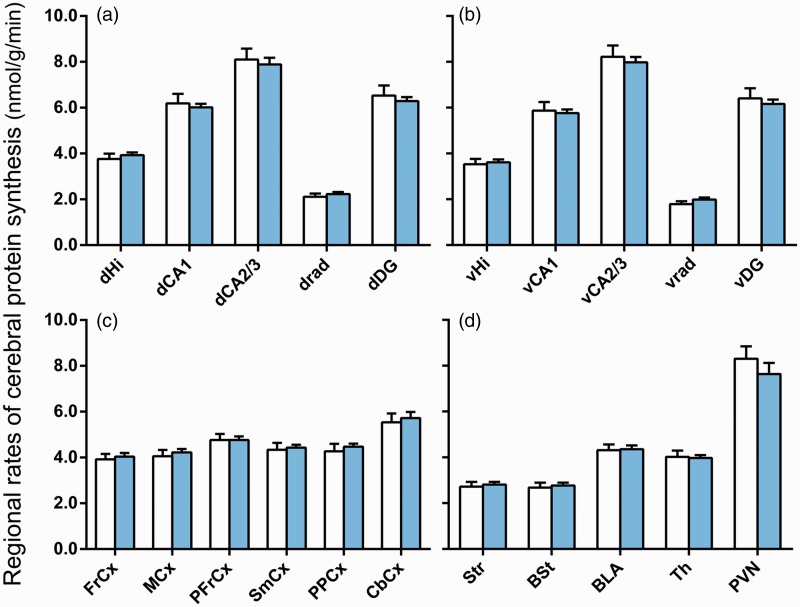
Regional rates of cerebral protein synthesis in WT (open bars) and KI_Dutch_ (filled bars) mice. Bars represent the means ± SEM for seven mice in each group except for the paraventricular nucleus of the hypothalamus with five WT mice and four KI mice. Data were analyzed by means of RM ANOVA with region as the within-subjects factor and genotype as the between-subjects factor. The interaction between region and genotype, *F*(2.7, 18.9) = 1.154, *p* = .35, was not statistically significant, and we found no statistically significant main effect of genotype, *F*(1, 7) = 0.676, *p* = .44. The main effect of region, *F*(2.7, 18.9) = 204.86, *p* < .001, was statistically significant. dHi = dorsal hippocampus; dCA1 = dorsal CA1 pyramidal cell layer; dCA2&3 = dorsal CA2&3 pyramidal cell layer; dRad = dorsal stratum radiatum; dDG = dorsal dentate gyrus; vHi = ventral hippocampus; vCA1 = ventral CA1 pyramidal cell layer; vCA2&3 = ventral CA2&3 pyramidal cell layer; vRad = ventral stratum radiatum; vDG = ventral dentate gyrus; FrCx = frontal association cortex; MCx = primary motor cortex; PFrCx = medial prefrontal cortex; SmCx = somatosensory cortex; PPCx = posterior parietal cortex; CbCx = cerebellar cortex; Str = striatum; BSt = bed nucleus of the stria terminalis; BLA = basolateral amygdala; Th = thalamus; PVN = paraventricular nucleus of the hypothalamus.

**Figure 3.**
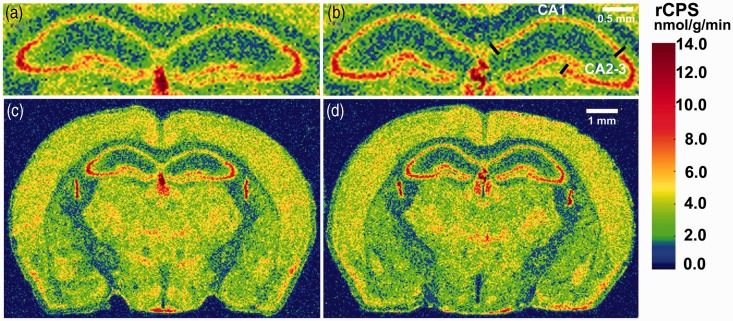
Digitized autoradiographic images color coded for rCPS at the level of dorsal hippocampus: (a and c) WT-C and (b and d) KI_Dutch_. Color bar applies to all four images. Scale bar in (b) (0.5 mm) applies to (a) and (b), and scale bar in (d) (1 mm) applies to (c) and (d). Images in (a) and (b) are enlarged subsections through the hippocampus taken from images in (c) and (d), respectively. Lines in (b) delineate the CA1 and the CA2-3 sectors of the pyramidal cell layer of the hippocampus. rCPS = regional rates of cerebral protein synthesis.

To compare the effects of the expanded CGG repeat in KI_Dutch_ mice to its effects in KI_NIH_ mice, we analyzed rCPS in the same 19 regions studied in our previous study with the addition of motor cortex and stratum radiatum of the dorsal and ventral hippocampus. In the present study, we determined rCPS in 21 brain regions including the dorsal hippocampus as a whole, the ventral hippocampus as a whole, four subregions of the dorsal and ventral hippocampus, six cortical areas, and five other regions (Figures 2 and 3). Results of rCPS determinations were analyzed by means of RM ANOVA with brain region as the within-subjects factor and genotype as the between-subjects factor. The interaction between region and genotype was not statistically significant, *F*(2.7, 18.9) = 1.154, *p* = .35, and there was no main effect of genotype, *F*(1, 7) = 0.676, *p* = .44. As expected from previous studies in which we measured rCPS *in vivo*, we did find a statistically significant effect of region, *F*(2.7, 18.9) = 204.86, *p* < .001, indicating that rCPS varies across brain regions. It is evident from the bar graphs (Figure 2) and the autoradiographs (Figure 3) that the mean values of rCPS in the two groups (WT and KI_Dutch_) are very similar.

## Discussion

The results of the present study show that in a mouse model of FXPM in which the CGG repeat length was between 120 and 159, *in vivo* rCPS measured in awake, behaving mice at 4 to 5 months of age were not different from those of WT control mice. Rates were normal despite a 50% decrease in brain FMRP levels. Our study suggests that there may be a threshold level of FMRP necessary for the maintenance of normal rates of protein synthesis.

We used the *in vivo* quantitative autoradiographic leucine method (Smith et al., 1988) to quantify rates of protein synthesis in our studies. With this methodology, we can measure steady-state rates of ongoing protein synthesis in conscious functioning mice. Measurements are average rates of leucine incorporation into all tissue proteins; our measurements do not target specific proteins. The relative contribution of an individual protein to the overall rCPS is weighted by both its fractional contribution to total tissue protein and its half-life in the tissue. The fact that we did not find increased rCPS in the KI_Dutch_ mice does not exclude the possibility that the synthesis of specific proteins may be affected, either increased or decreased. One of the advantages of the autoradiographic method is that it enables us to measure rCPS in all regions of the brain. The spatial resolution of the autoradiographic method is 50 µm (Smith, 1983). This permits us to distinguish specific nuclei and cell layers, including dendritic-rich and cell body layers. We cannot, however, distinguish specific cell types within a layer.

The absence of FMRP is at the biochemical core of FXS. FMRP is a polyribosome-associated RNA-binding protein, suggesting that it may play a role in regulating translation. In *in vitro* model systems (Laggerbauer et al., 2001; Li et al., 2001), FMRP binds its target mRNAs and negatively regulates their translation. FMRP has been shown to reversibly stall ribosomes specifically on its target mRNAs during elongation (Darnell et al., 2011). FMRP also recruits cytoplasmic FMRP-interacting protein (CYFIP1) to block formation of the eIF4F complex preventing translation initiation (Napoli et al., 2008). FMRP also can recruit RNA-induced silencing complex (RISC) to inhibit translation (Caudy et al., 2002). Consistent with this role for FMRP as a suppressor of translation, elevated rates of protein synthesis measured in the intact nervous system have been demonstrated in *Fmr1* KO mice (Qin et al., 2005). The most affected regions were hippocampus, hypothalamus, thalamus, amygdala, and frontal and parietal cortex. Studies in hippocampal slices from *Fmr1* KO mice confirm increased incorporation rates (Dölen et al., 2007). This core pathophysiological change in the disease might be used as a biomarker of effective drug treatment (Liu et al., 2012; Michalon et al., 2012).

We were interested in knowing whether changes in rCPS occur in FXPM because of reports that patients with FXPM have symptoms suggesting a milder form of FXS (Cornish et al., 2005; Hunter et al., 2008; Kogan et al., 2008; Cornish et al., 2009) and because FMRP concentrations in lymphocytes may be decreased in these patients (Tassone et al., 2000). We hypothesized that with reduced concentrations of FMRP, rCPS might be increased but to a lesser extent than seen in *Fmr1* KO mice. In our previous study of the KI_NIH_ mouse model of FXPM with CGG repeat lengths between 120 and 140, we found that FMRP levels in brain were reduced by 80% to 90% of WT and rCPS were increased (106–120% of WT) similar to changes we had seen in *Fmr1* KO mice. KI_NIH_ animals also had a behavioral phenotype comparable to *Fmr1* KO mice. In the KI_Dutch_ mouse model, we selected mice with CGG repeat lengths between 120 and159 because animals with repeat lengths in this range had FMRP levels about 50% of WT. The effect that we found on FMRP levels was similar to previous reports in this model. In KI_Dutch_ mice with CGG_(100–150)_, a 40% decrease in brain FMRP (*p* = .11) was reported in 1-year-old animals (Brouwer et al., 2008a). In KI_Dutch_ mice with CGG_(84–210)_, *Fmr1* mRNA expression was increased in brain by approximately 1.4 fold compared with WT mice (*p* < .05), whereas brain levels of FMRP were reduced by 28% (Berman et al., 2012).

Controlling for CGG repeat length, background strain, and age of the animals, the two FXPM mouse models, KI_Dutch_ and KI_NIH_, yield similar results with respect to changes in *Fmr1* mRNA levels and changes in dendritic structure, but with respect to rCPS and behavioral phenotype, the two models diverge. In the KI_Dutch_ model, *Fmr1* mRNA levels in brain were increased threefold over WT (Willemsen et al., 2003). Increases were similar in the KI_NIH_ model, two- to fourfold in most brain regions (Qin et al., 2011). Compared with WT mice, KI_Dutch_ mice showed fewer dendritic branches proximal to the soma, reduced total dendritic length, and a higher frequency of longer dendritic spines (Berman et al., 2012). KI_NIH_ mice showed reduced dendritic complexity and increased dendritic spine length in pyramidal neurons in medial frontal cortex, hippocampus, and basal amygdala (Qin et al., 2011). In the present study, we did not determine the behavioral phenotype of KI_Dutch_ animals. Others have shown that KI_Dutch_ mice (CGG repeat length 106–123) are not impaired on the Morris water maze, passive avoidance test, open field test, and tests of neuromotor performance at 140 days of age (Van Dam et al., 2005), although there are some deficits on the Morris water maze and open field tests that occur as the animals age. Further, tests of KI_Dutch_ mice with CGG repeat lengths between 80 and 180 (Hunsaker et al., 2009) indicate some subtle deficits in spatial information processing at 84 and 168 days of age that become more extensive in older animals. Young adult KI_NIH_ mice had a very similar behavioral phenotype to *Fmr1* KO mice (Qin et al., 2011), with hyperactivity, reduced anxiety, impaired social interactions, and deficits on passive avoidance test of learning and memory.

The reason for decreased levels of FMRP in the presence of excess *Fmr1* mRNA is not understood, but it is thought that transcripts with expanded repeats may impede the linear 40 S migration along the 5′-untranslated region (Feng et al., 1995). The similarities in the expanded repeat sequences and *Fmr1* mRNA levels in the two models, however, cannot explain the difference in FMRP levels. Moreover, in the generation of both models, the *neo* cassette was removed by crossing KI mice with mice expressing *Cre* recombinase, so the changes to *Fmr1* were minimal in both models. It has recently been reported that the KI_NIH_ model, but not the KI_Dutch_ model, retains a stop codon 18 bp before the CGG repeat (Todd et al., 2013). Consistent with this difference, FMRpolyG is produced in KI_Dutch_ mice likely through a repeat-associated non-AUG-initiated (RAN) translation mechanism. How the synthesis of FMRpolyG might positively influence FMRP levels is not clear. One proposal is that translation through the repeat may facilitate *Fmr1* translation by assisting RNA unwinding by means of helicase recruitment (Todd et al., 2013). With CGG repeat lengths in the normal range, the polyglycine peptides produced in this process may be readily cleared from cells, but expanded repeats (50–200 CGG) produce larger polyglycine proteins that with time accumulate in the cell and are cytotoxic. The decreased levels of FMRP and the absence of FMRpolyG in the KI_NIH_ may suggest that the RAN translation mechanism is important for translation of *Fmr1* in the presence of an expanded CGG repeat sequence.

The present and previous studies of the KI_Dutch_ model (Brouwer et al., 2008a; Berman et al., 2012) are in accord with each other with respect to FMRP levels in brains. Our current study suggests that FMRP at 50% of WT levels is sufficient for normal cerebral protein synthesis rates and what appears to be normal brain function in young adult mice.

## Conclusions

Our study of the adult male KI_Dutch_ mouse model of FXPM with 120 and 159 CGG repeats showed that *in vivo* rCPS were normal despite a 50% decrease in brain FMRP levels. These results suggest that there may be a threshold level of FMRP necessary for the maintenance of normal rates of protein synthesis in the nervous system. As animal’s age and FMRP levels in brain decline, further symptoms may unfold.

## Summary

In a fragile X premutation mouse model, regional rates of cerebral protein synthesis rates (rCPS) were normal despite 50% fragile X mental retardation protein (FMRP) reductions. Results suggest a threshold level of FMRP may be necessary for maintenance of rCPS. With aging and reduced FMRP, symptoms may unfold.
